# Knockdown resistance mutations in *Phlebotomus argentipes* sand flies in Bihar, India

**DOI:** 10.1186/s13071-024-06424-0

**Published:** 2024-08-09

**Authors:** Mojca Kristan, Carlamarita Hazelgrove, Kundan Kumar, Ashish Kumar, Vijay Kumar, Pradeep Das, Emma Collins, Miguella Mark-Carew, Susana Campino, Mary Cameron

**Affiliations:** 1 Department of Disease Control, London School of Hygiene & Tropical Medicine, London, UK; 2 Rajendra Memorial Research Institute of Medical Sciences, Patna, India; 3 Department of Molecular Parasitology, National Institute of Cholera and Enteric Diseases, Kolkata, India; 4 Department of Infection Biology, London School of Hygiene & Tropical Medicine, London, UK

**Keywords:** Visceral leishmaniasis, *Phlebotomus argentipes*, Vector surveillance, Insecticide resistance, *kdr* mutations

## Abstract

**Background:**

Vector control based on indoor residual spraying (IRS) is one of the main components of the visceral leishmaniasis (VL) elimination programme in India. Dichlorodiphenyltrichloroethane (DDT) was used for IRS until 2015 and was later replaced by the synthetic pyrethroid alpha-cypermethrin. Both classes of insecticides share the same target site, the voltage-gated sodium channel (*Vgsc*). As high levels of resistance to DDT have been documented in the local sand fly vector, *Phlebotomus argentipes*, it is possible that mutations in the *Vgsc* gene could provide resistance to alpha-cypermethrin, affecting current IRS pyrethroid-based vector control.

**Methods:**

This study aimed to compare frequencies of knockdown resistance (*kdr*) mutations in *Vgsc* between two sprayed and two unsprayed villages in Bihar state, India, which had the highest VL burden of the four endemic states. Across four villages, 350 female *P. argentipes* were collected as part of a 2019 molecular xenomonitoring study. DNA was extracted and used for sequence analysis of the IIS6 fragment of the *Vgsc* gene to assess the presence of *kdr* mutations.

**Results:**

Mutations were identified at various positions, most frequently at codon 1014, a common site known to be associated with insecticide resistance in mosquitoes and sand flies. Significant inter-village variation was observed, with sand flies from Dharampur, an unsprayed village, showing a significantly higher proportion of wild-type alleles (55.8%) compared with the three other villages (8.5–14.3%). The allele differences observed across the four villages may result from selection pressure caused by previous exposure to DDT.

**Conclusions:**

While DDT resistance has been reported in Bihar, *P. argentipes* is still susceptible to pyrethroids. However, the presence of *kdr* mutations in sand flies could present a threat to IRS used for VL control in endemic villages in India. Continuous surveillance of vector bionomics and insecticide resistance, using bioassays and target genotyping, is required to inform India’s vector control strategies and to ensure the VL elimination target is reached and sustained.

**Graphical Abstract:**

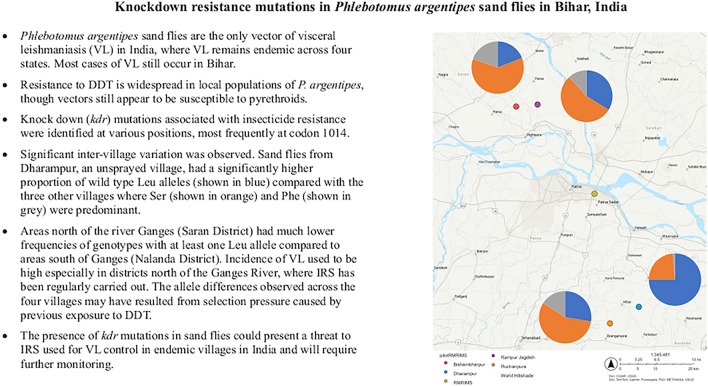

## Background

Vector-borne diseases (VBDs) account for around 17% of the estimated global burden of all infectious diseases, disproportionately affecting poorer populations in tropical and subtropical areas [1]. Visceral leishmaniasis (VL), caused by parasites of the *Leishmania donovani* complex, and transmitted by phlebotomine sand flies, is responsible for an estimated 30,000 cases each year with high fatality rates if untreated. In 2023, 80 countries were endemic for VL, with most cases occurring in three eco-epidemiological hotspots, namely East Africa (72% of total cases), Brazil (13%) and the Indian subcontinent (8%) [2]. Significant progress in controlling VL has been made, especially in the Indian subcontinent, where an elimination initiative was launched in 2005. Bangladesh already met the elimination threshold (< 1 detected VL case per 10,000 population per year at the upazila level) in 2017 and has sustained elimination since then. In 2022, in India and Nepal the VL programme target was reached in 99% of endemic implementation units (blocks in India and districts in Nepal) [2–7].

In India, VL remains endemic across four states: Bihar, Jharkhand, Uttar Pradesh and West Bengal. It caused 818 cases and 3 deaths in 2022, with most cases still occurring in Bihar [8]. The only VL vector in India is *Phlebotomus argentipes*, considered a primarily endophilic and endophagic species [9, 10]. Hence, vector control by indoor residual spraying (IRS) has been one of the main components of VL elimination programme, exploiting sand fly bionomics and also building on the past experience of malaria control IRS additionally resulting in VL control [11]. However, reports from Bihar indicate this species may be more exophilic and exophagic than previously thought and is an opportunistic feeder [12–14], indicating that IRS might need to be supplemented with other vector control tools [13, 15].

The accelerated plan for VL elimination in India recommends two rounds of IRS in endemic villages [16]. DDT was used for IRS until 2015 when it was replaced by the synthetic pyrethroid alpha-cypermethrin [17] because of the widespread DDT resistance of *P. argentipes* documented in many regions, including the state of Bihar [18–23]. This vector appears to remain susceptible to pyrethroids, with only minimal possible resistance detected to alpha-cypermethrin, based on mortality ranging between 97.6 and 100% [21, 24].

DDT and alpha-cypermethrin target the voltage-gated sodium channel (*Vgsc*). Knockdown resistance mutations (*kdr*) within the *Vgsc* are a major mechanism of resistance against DDT and pyrethroids in many insects, including mosquitoes and sand flies [25, 26]. Wild-type *Vgsc* is normally a leucine residue (Leu or L), with L1014F and L1014S being the most common mutations in insects, where Leu changes to phenylalanine (Phe or F) or serine (Ser or S) [25, 27, 28]. Both L1014F and L1014S have been detected in sand flies in Bihar previously [28] and were also already detected in Sri Lanka [29]. Apart from codon 1014, other codons associated with insecticide resistance were detected in *Aedes aegypti* mosquitoes (codons 1011 and 1016) and in other insect orders besides Diptera such as Lepidoptera and Blattodea (codon 1020) [28].

A better understanding of insecticide resistance in sand fly populations is needed to improve the efficiency of vector control and achieve sustained elimination of VL. Rotation of insecticides to prevent emergence of insecticide resistance, together with susceptibility testing to inform insecticide choice, has already been recommended [15]. The aim of the present study was to compare the frequencies of *kdr* mutations in *Vgsc*, particularly at codon 1014, but also codons 1011 and 1016, between the two VL endemic sprayed and two unsprayed villages in Bihar, India.

## Methods

### Sand fly collections

**Fig. 1:**
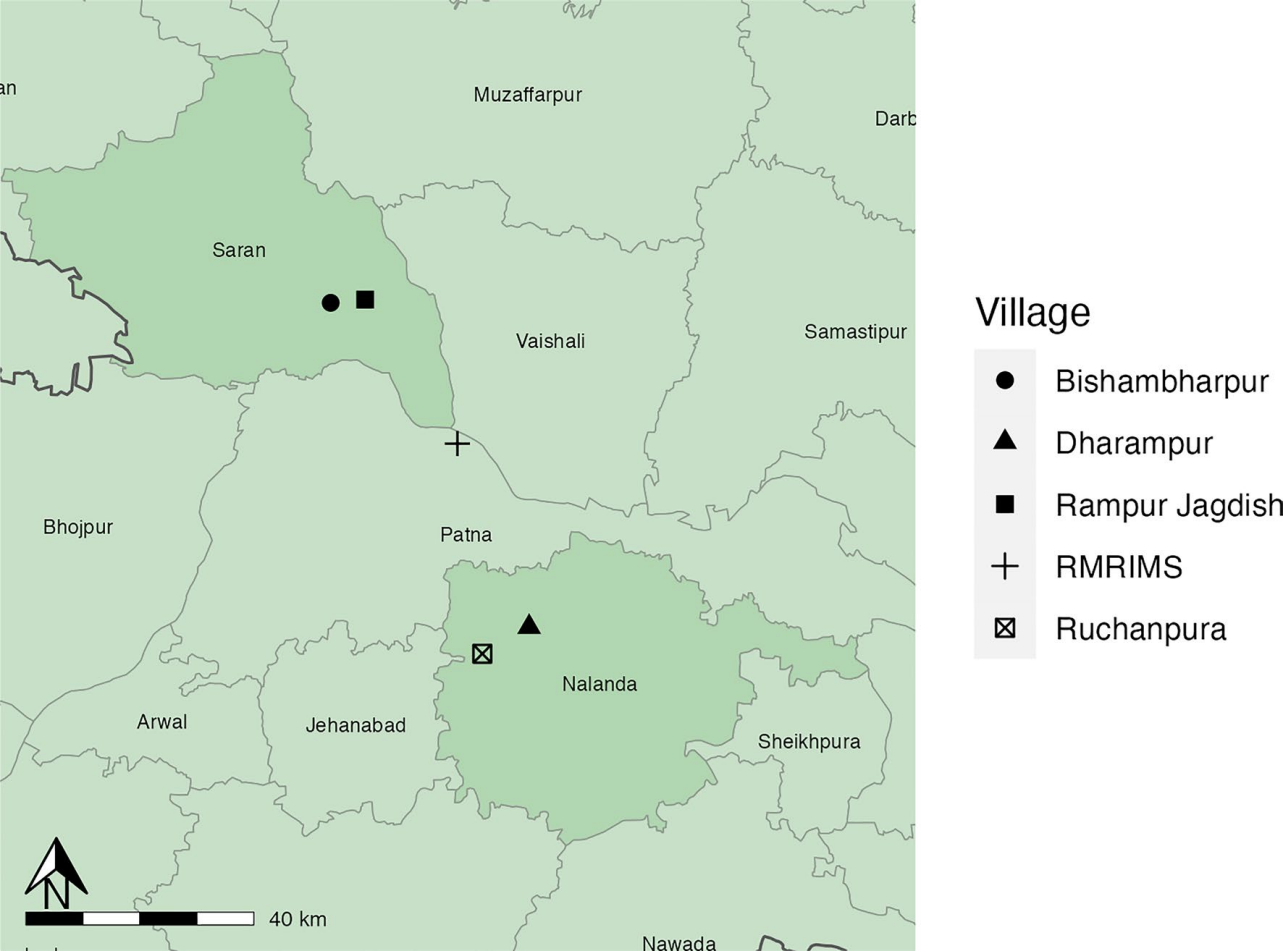
A map of the study area, showing the endemic villages in Saran District and control villages in Nalanda District. Map generated using R software

*Phlebotomus argentipes* females were sampled in a previous study, comparing CDC light traps, mechanical vacuum aspirators and Prokopack aspirator collection methods. The study was performed in May–June 2019 in four villages in Bihar: Rampur Jagdish and Bishambharpur in Saran District where VL was endemic and Dharampur and Ruchanpura in Nalanda District with no VL transmission [30] (Fig. 1).

Rampur Jagdish was sprayed in May 2018 and May and September of 2019, while Bishambharpur was sprayed in April 2018 and April and September of 2019. Dharampur and Ruchanpura were not sprayed.

### Genotyping of *kdr* mutations

Three hundred fifty *P. argentipes* females were used for sequence analysis of the IIS6 fragment of the *Vgsc* gene. DNA from the samples was extracted using Qiagen DNeasy Blood & Tissue Kits following the manufacturer’s protocol [30] and quantified using a NanoDrop (Thermofisher). The *Vgsc* primers (Vssc8F: 5ʹ ± AATGTGGGATTGCATGCTGG ± 3ʹ, Vssc1bR: 5ʹ ± CGTATCATTGTCTGCAGTTGGT ± 3ʹ) described by Gomes et al. [28] were used to amplify a genomic DNA fragment from *VGSC* domain II, segment 6, which includes codons 1011, 1014 and 1016.

A volume of 2 µl of sample DNA (around 1–5 ng of DNA) was used per reaction. Polymerase chain reaction (PCR) was carried out in a total volume of 25 µl by combining 5 µl of 5 × Q5 reaction buffer (NEB), 1.25 µl of each forward and reverse primer (10 µM stock), 0.5 µl of 10 mM dNTPs, 14.75 µl of nuclease free water and 0.25 µl of Q5 DNA polymerase enzyme (NEB), as per protocol (New England Biolabs Inc.). Thermocycling conditions followed those described by Gomes et al. [28] (initial denaturation step of 5 min at 95 °C, followed by 30 cycles each of 96 °C for 30 s, 56 °C for 30 s and 72 °C for 30 s, and a final extension step of 72 °C for 5 min), using G- Storm Thermal Cycler PCR machine.

The quality of PCR products (5 µl each) was checked using 1% agarose gel electrophoresis in TAE buffer, stained with Sybr Safe (ThermoFisher) and visualised on a BIO-RAD ChemiDoc MP Imaging System. Bands visualised at around 400 base pairs indicated that the fragment was correctly amplified.

### Sanger sequencing

The PCR products were sent for Sanger sequencing (Genewiz, Takeley, Essex, UK), obtaining forward and reverse sequence data for each sample. Genetic sequence data were visualised, trimmed and edited using SnapGene Viewer (version 5.3.2, 2022). Both forward and reverse sequences for each sample were analysed. Sequences from FASTA format files were aligned using Aliview software (version 1.23) and mutations recorded. BLASTn tool from the National Centre for Biotechnology Information (NCBI) was used to confirm the taxonomic identification. Databases to which sequences were compared were sourced from GenBank (accession nos. KY114615, KY114616, KY114617, KY114618, KY114619) [28].

### Mutation profiling

Samples that were Leu homozygotes (wild-type) or Leu/Ser or Leu/Phe heterozygotes could be classified as organochlorine (i.e. DDT) susceptible, whereas those with *kdr* genotypes (Ser/Ser and Phe/Phe homozygotes or Ser/Phe heterozygotes) could potentially be classified as resistant, in line with previous findings by Gomes et al. [28] where allele frequencies in female sand flies that survived or were killed by insecticide exposure were compared.

Frequencies of alleles that could be identified as Leu, Ser or Phe from the genetic sequence data were calculated based on the total number of alleles in each village. Chi-square tests were used to assess differences between sand flies in allele and genotype frequencies at *Vgsc* codon 1014 per village. For smaller sample sizes, Fisher’s exact tests were used to obtain exact *P* values.

### Accession numbers

All sequences were submitted to the European Nucleotide Archive (ENA) (project number PRJEB74041; accession nos. ERR12764362–ERR12764733).

## Results

The sequence analysis of the IIS6 fragment of the *Vgsc* gene was carried out for 350 samples of *P. argentipes* collected in sprayed and unsprayed villages in Bihar. Three hundred ten samples (out of 350) were of sufficient quality to be included in further data analysis: 77 from Dharampur and 42 from Ruchanpura, the two unsprayed villages from Nalanda; 85 from Rampur Jagdish and 106 from Bishambharpur, the two sprayed villages from Saran. Mutations were identified at various positions within the IIS6 fragment, most frequently at codon 1014, causing replacement of the wild type leucine codon (TTA) with serine, L1014S (TCA/TCT/TCC) or leucine with phenylalanine, L1014F (TTC and TTT). The most frequent were mutations resulting in the L1014S codon (48.5%), followed by wild type L1014 codon (39.5%). Additionally, mutations causing replacement L1014F were present in almost 12% of specimens (Table 1).

**Table 1:** Overall allele frequencies at codon 1014

Amino acid	Allele	Frequency (%)
Leu	TTA	39.5
Ser	TCA	43.3
Ser	TCT	3.9
Ser	TCC	1.3
Phe	TTT	7.1
Phe	TTC	4.9

**Fig. 2:**
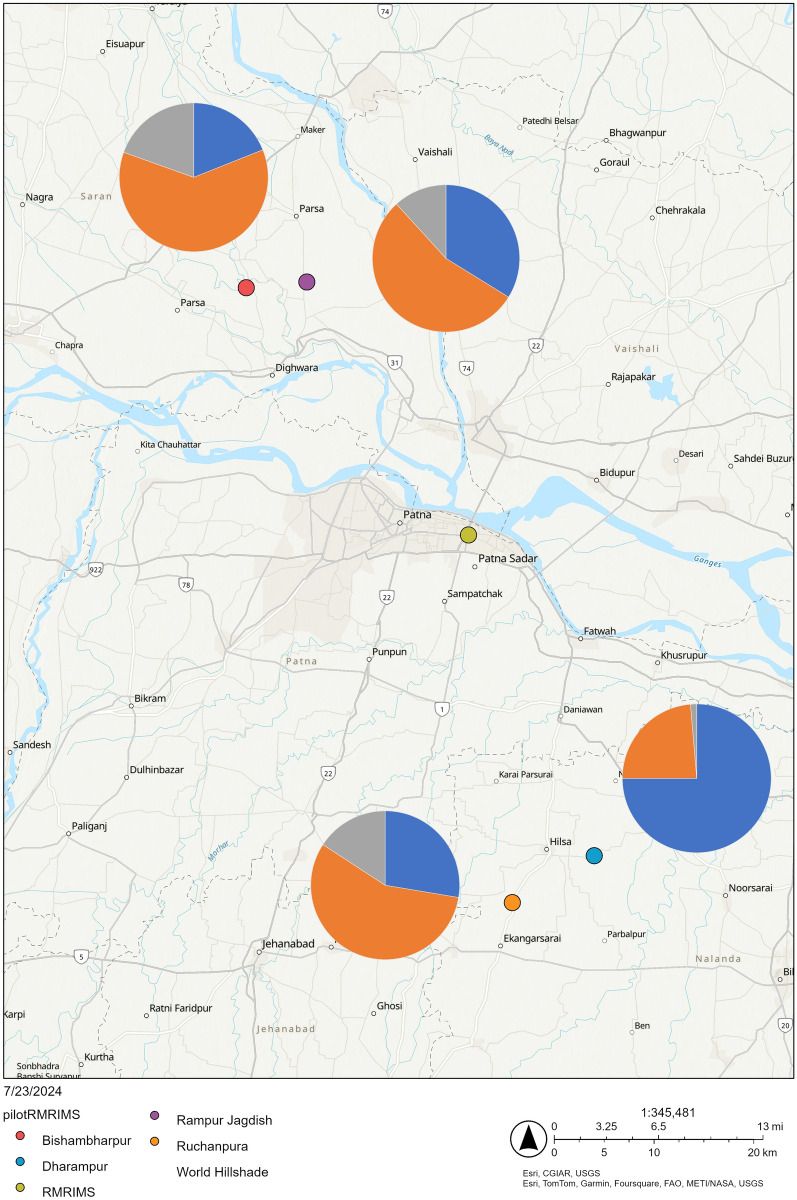
A map showing locations of villages where samples were collected, together with *Vgsc-*1014 allele frequencies (pie chart colours represent different alleles: Leu = blue, Ser = orange, Phe = grey). Base layer available at World Topo Map (MapServer) (arcgisonline.com). Map generated using Epi Info 7 software

Significant inter-village variation in allele and genotype frequencies was observed. Sand flies from Dharampur, an unsprayed village, had a significantly higher proportion of wild-type homozygotes (55.8%) compared to the three other villages (8.5–14.3%), whereas almost 30% of sand flies in this location were Leu/Ser heterozygotes (Tables 2 and 3; Fig. 2) (allelic inter-village comparison $\chi_{6}^{2}$ = 105.7, *P* = 1.6 × 10^–20^; genotypic comparison of wild-type homozygotes vs. Leu heterozygotes vs. other genotypes, $\chi_{6}^{2}$ = 99.8, *P* = 2.8 × 10^–19^). The other three villages, including unsprayed Ruchanpura, had a high frequency of samples with L1014S mutation and a mixture of different heterozygotes.

**Table 2:** Information on sampling sites and allele frequencies at codon 1014 of *Vgsc*

District	Village	IRS	Latitude	Longitude	*N*	Leu	Ser	Phe
Nalanda	Dharampur	No	25.31119197	85.32337202	154	0.721	0.227	0.013
	Ruchanpura	No	25.269017	85.242599	84	0.250	0.512	0.222
Saran	Rampur Jagdish	Yes	25.8275441	85.03642289	170	0.271	0.435	0.094
	Bishambharpur	Yes	25.82298536	85.01153214	212	0.156	0.505	0.160

IRS: information on whether IRS was used in the village or not;* N*: total number of alleles in each village; frequencies only provided for those that could be identified as Leu, Ser or Phe; Leu: leucine, which is a wild-type allele found in insecticide-susceptible sand flies

**Table 3:** Genotype frequencies at codon 1014 in different villages

Village/District	N	Leu/*			Total	Ser/Ser	Ser/Phe	Phe/Phe	Other
		Leu/Leu	Leu/Ser	Leu/Phe					
Dharampur	77	43	23	2	68 (88.3)	6 (7.8)	0	0	3 (3.9)
Ruchanpura	42	6	7	2	15 (35.7)	15 (35.7)	6 (14.3)	2 (4.8)	4 (9.5)
Nalanda District	119	49	30	4	83 (69.7)	21 (17.6)	6 (5.0)	2 (1.7)	7 (5.9)
Rampur Jagdish	85	10	21	5	36 (42.3)	26 (30.6)	1 (1.2)	5 (5.9)	17 (20.0)
Bishambharpur	106	9	15	0	24 (22.6)	41 (38.7)	10 (9.4)	12 (11.3)	19 (17.9)
Saran District	191	19	36	5	60 (31.4)	67 (35.1)	11 (5.8)	17 (8.9)	36 (18.8)

Values in brackets are relative frequencies (percentage)

Leu/*: number of genotypes that include at least one wild-type leucine (Leu) allele; Ser/Ser: number of individuals homozygous for serine; Ser/Phe: number of heterozygotes with serine and phenylalanine; Phe/Phe: number of homozygotes for phenylalanine; Other: heterozygotes with serine and either leucine or phenylalanine (TYM or TYW)

There was also significant allelic and genotypic variation between the unsprayed Nalanda and sprayed Saran districts (Tables 2 and 3, Fig. 2) (allelic $\chi_{3}^{2}$ = 62.3, *P* = 2.98 × 10^–14^; genotypic $\chi_{4}^{2}$ = 42.5, *P* = 1.3 × 10^–8^). Whereas 41% of samples from Nalanda were of wild-type genotypes (Leu/Leu) and further 29% were heterozygotes with one susceptible Leu allele, most samples from Saran (68.6%) entirely lacked the susceptible allele.

## Discussion

Knowledge on insecticide resistance mechanisms in phlebotomine sand flies is limited [30], even though vector control is a key component of leishmaniasis control and elimination in the absence of a vaccine and where there are difficulties related to diagnosis and treatment [6, 30, 31]. Our report on the presence of *kdr* mutations in the *Vgsc* gene in *P. argentipes* sand flies from Bihar, India, adds to a very limited number of *kdr* reports from the Indian subcontinent [28, 29, 32, 33].

Like in mosquitoes, insecticide resistance in sand flies can be caused by target site mutations, such as *kdr* mutations in the *Vgsc* gene, or it can be metabolic, where resistant insects have higher levels or more efficient forms of the enzymes that cause degradation or sequestration of insecticides; thus far, no reports on cuticular or behavioural resistance mechanisms in sand flies are available [34]. Furthermore, fewer studies report on metabolic resistance.

Spatial and temporal variation in *kdr* allele frequencies has been described in *Anopheles* [35, 36] and *Aedes* mosquitoes [37] as well as sand flies [33]. A high frequency of *kdr* mutations, both L1014S and L104F, was observed in this study, similar to previously reported data [28, 33]. There was significant inter-village variation in allele frequencies: sand flies from Dharampur, an unsprayed village, had more wild-type Leu alleles than other villages—both unsprayed Ruchanpura and especially the two villages from Saran where IRS takes place. These observed differences across the four villages might have resulted at least partially from selection pressure caused by insecticide exposure in the past, as other studies have shown evidence for links between past IRS exposure and *kdr* frequencies [33]. *Kdr* mutations L1014S and L1014F are associated with insecticide resistance—specifically resistance to organochlorines such as DDT and to pyrethroids—in many vectors [38, 39], including sand flies [25, 27, 40]. DDT has been used for IRS in India since the 1950s and was replaced by the pyrethroid alpha-cypermethrin in 2015. While DDT resistance has been reported in Bihar, *P. argentipes* is still susceptible to pyrethroids [18, 19, 22, 28, 41]. DDT resistance can also be caused by glutathione S-transferases (GSTs) [42]. The involvement of this metabolic resistance mechanism was already observed in *P. argentipes* in Bihar [43] and also in *P. argentipes* populations from Sri Lanka [28]. Furthermore, pyrethroid resistance has already been detected in sprayed and also unsprayed villages in Nepal [44].

We compared our findings with other data from this area [28] where a lot of variation in the frequency of genotypes between different districts was also observed. Dharampur (Nalanda District), a village from this study with the largest proportion of wild-type sand flies, is close to Dhanarua (Patna District), an area with the largest proportion of wild-type sand flies in the study carried out by Gomes et al. [28]. In both studies, areas north of the river Ganges (Saran District and Vaishali District) had much lower frequencies of genotypes with at least one Leu allele compared to areas south of Ganges (Nalanda District and Patna District). Incidence of VL used to be high especially in districts north of the Ganges River, such as Vaishali and Saran, where IRS has been regularly carried out [45–47].

Data on insecticide resistance on the Indian subcontinent obtained from bioassays have been reviewed by Dhiman and Yadav [48]. The presence of *kdr* mutations in *P. argentipes* in Bihar, India, linking the presence of mutations to phenotypic data, was first described in 2017 [28]. While both L1014S and L1014F confer resistance to DDT, it is thought that L1014F confers stronger resistance to DDT than L1014S, and only two mutant alleles (Phe/Phe, Phe/Ser and Ser/Ser) are thought to produce a resistant phenotype [28]. Phenylalanine frequencies as reported from Vaishali and also Patna Districts [28] were much higher than those we observed in Saran in our study (53.9% and 29.0% vs. 13.1%).

The main limitations of our study are the lack of bioassay data and having no knowledge of the resistant phenotypes present in the area. Repeated sampling and testing would help show if and how *kdr* frequencies are changing and whether resistance is increasing, including resistance to pyrethroids. Repeated sampling across the years in eight sentinel sites in VL-endemic areas in northeastern India showed some temporal variations in the frequencies of the *kdr* genotypes, resulting in both increases and decreases between the years [33].

## Conclusions

The presence of *kdr* in sand flies could present a threat to IRS used for VL control in endemic villages in India. Use of different insecticides, such as carbamates, organophosphates or neonicotinoids, as well as alternative or supplemental methods of control, should be explored for the purpose of insecticide resistance management and potential integration of vector control activities for VL and other vector-borne diseases [34, 49, 50]. Further research on vector bionomics and insecticide resistance will be required to inform India’s vector control strategies and ensure the VL elimination target is reached and sustained—including pairing of bioassay data to confirm the resistance phenotype of sand fly populations with genotypic data and transcription levels of metabolic enzymes that cause resistance to insecticides.

## Data Availability

All data supporting the findings of this study are available within the paper, while DNA sequences have been deposited in the European Nucleotide Archive (ENA), with the primary accession code (project number) PRJEB74041.
